# 

**Published:** 1859-09

**Authors:** 


### LIST OF COLLABORATORS.

---

S. G. Armor, M.D., late Professor of Pathology and Clinical Medicine, Missouri Med. College.
John L. Atlee, M.D., late President Pennsylvania State Medical Society.
Walter F. Atlee, M.D., Philadelphia.
Robert G. Barclay, M.D., Jerusalem, Syria.
John Bell, M.D., Philadelphia, formerly Professor of Medicine, Medical College of Ohio.
T. S. Bell, M.D., Professor of Medicine, University of Louisville.
S. M. Bemiss, M.D., Professor of Clinical Medicine, University of Louisville.
L. P. Blackburn, M.D., New Orleans, La.
George C. Blackie, M.D., Professor of Botany, University of Nashville.
G. C. Blackman, M.D., Professor of Surgery, Medical College of Ohio.
J. L. Bobbs, M.D., formerly Professor of Anatomy, Indianapolis.
W. S. Bowen, M.D., New York.
N. Bozeman, M.D., Montgomery, Ala.
J. T. Bradford, M.D., Augusta, Ky.
John H. Brinton, M.D., Lecturer on Operative Surgery, Philadelphia.
W. S. Chipley, M.D., Professor of Medicine, Transylvania University.
A. Clapp, M.D., New Albany, Ia.
D. B. Cliffe, M.D., Franklin, Tenn.
J. Da Costa, M.D., Lecturer on the Practice of Medicine at the Philad. Med. Association.
Samuel H. Dickson, M.D., Professor of Medicine, Jefferson Medical College.
M. Dupierris, M.D., Havana, Cuba.
Richard J. Dunglison, M.D., Philadelphia.
W. F. Edgar, M.D., United States Army.
S. C. Farrar, M.D., Jackson, Miss.
C. S. Fenner, M.D., Memphis, Tenn.
Emil Fischer, M.D., Philadelphia.
Austin Flint, M.D., Professor of Clinical Medicine, Auscultation and Percussion, New Orleans School of Medicine.
Charles Frick, M.D., Baltimore.
W. Y. Gadbury, M.D., Benton, Miss.
O. C. Gibbs, M.D., Chautauque County, New York.
Dr. J. P. Hall, Glasgow, Ky.
William A. Hammond, M.D., United States Army.

JOHN HARDIN, M.D., Professor of Obstetrics, Kentucky School of Medicine, Louisville.

EDWARD HARTSHORNE, M.D., One of the Surgeons to Wills Hospital for Diseases of the Eye, Philadelphia.

E. B. HASKINS, M.D., Professor of Chemistry, Shelby Medical College, Nashville, Tenn.

C. A. HENTZ, M.D., Marianna, Florida.

ADDINELL HEWSON, M.D., One of the Surgeons to Wills Hospital for Diseases of the Eye, Philadelphia.

SAMUEL L. HOLLINGSWORTH, M.D., late Editor of the Medical Examiner, Philadelphia.

WILSON JEWELL, M.D., Philadelphia, President of the Quarantine and Sanitary Convention.

WM. V. KEATING, M.D., Lecturer on Obstetrics and the Diseases of Women, at the Philadelphia Medical Association.

JOHN G. KERR, M.D., Canton, China.

G. A. KUNKLER, M.D., Madison, Ia.

BEN. LOGAN, M.D., Shreveport, La.

A. LOPEZ, M.D., Mobile, Ala., formerly President of the Alabama State Medical Society.

J. AITKEN MEIGS, M.D., Professor of the Institutes of Medicine in the Medical Department of Pennsylvania College.

S. WEIR MITCHELL, M.D., Lecturer on Physiology at the Philad. Med. Association.

GEORGE R. MOREHOUSE, M.D., Philadelphia.

B. I. RAPHAEL, M.D., New York.

J. C. REEVE, M.D., Dayton, Ohio.

L. C. RIVES, M.D., formerly Professor of Midwifery, Medical College of Ohio.

J. LAWRENCE SMITH, M.D., Professor of Chemistry, University of Louisville.

W. C. SNEED, M.D., Frankfort, Ky., late President Kentucky State Medical Society.

C. H. SPILMAN, M.D., Harrodsburg, Ky., late President of Kentucky State Medical Society.

GEO. SUTTON, M.D., Aurora, Ia.

W. L. SUTTON, M.D., Georgetown, Ky., formerly President Kentucky State Medical Society.

HORATIO R. STORER, M.D., formerly one of the Physicians to the Boston Lying-in Hospital, Boston, Mass.

S. THOMPSON, M.D., Albion, Ill., late President of the Illinois State Medical Society.

E. WILLIAMS, M.D., Cincinnati, Ohio.

### Bi-Monthly Bibliographical Record.

### AMERICAN.

A System of Surgery: Pathological, Diagnostic, Therapeutic, and Operative. By S. D. Gross, M.D., Professor of Surgery in the Jefferson Medical College of Philadelphia, etc. 2 vols. 8vo., pp. 1162–1198. Philadelphia: Blanchard & Lea, 1859. (From the publishers.)

Woman; her Diseases and Remedies. A Series of Letters to his Class. By Charles D. Meigs, M.D., Professor of Midwifery and the Diseases of Women and Children in the Jefferson Medical College of Philadelphia, etc. 4th edition. 8vo., pp. 706. Blanchard & Lea: Philadelphia, 1859. (From the publishers.)

The Virginia Springs, and Springs of the South and West. By T. J. Moorman, M.D., etc. 12mo., pp. 403. J. B. Lippincott & Co.: Philadelphia, 1859. (From the publishers.)

Contributions to Midwifery, and Diseases of Women and Children, with a Report on the Progress of Obstetrics, and Uterine and Infantile Pathology in 1858. By E. Noeggerath, M.D., and A. Jacobi, M.D. 8vo., pp. 466. New York: Ballière Brothers, 1859. (From the authors.)

Physicians' Visiting List, Diary, and Book of Engagements, for 1860. Philadelphia: Lindsay & Blakiston. (From the publishers.)

Proceedings of the Sixty-Seventh Annual Convention of the Connecticut Medical Society, held at Middletown, May 25th and 26th, 1859. 8vo., pp. 106. (From the secretary.)

Medical Heroism. Address delivered before the Philadelphia County Medical Society, February 24th, 1859. By John Bell, M.D. 8vo., pamphlet, pp. 38. (From the author.)

Some Account of a Secret Society in New York, entitled the "Kappa-Lambda." In a letter to Alexander Stevens, M.D., by a Retiring Physician. Pamphlet, 8vo., pp. 37. 1859. (From the author.)

Medical Communications of the Massachusetts Medical Society. Vol. ix. No. 5. 8vo., pp. 130. Boston, 1859. (From the Society.)

Reminiscences of Samuel Latham Mitchill, M.D., LL.D. By John W. Francis, M.D., etc. Pamphlet, 8vo., pp. 31. New York, 1859. (From the author.)

Experimental Researches Relative to Corroval and Vao. By William A. Hammond, M.D., and S. Weir Mitchell, M.D. Reprint, pp. 48. Philadelphia, 1859. (From the authors.)

Rational Medicine and Thomsonianism. By T. Dickson Smith, M.D., of Macon, Georgia. 8vo., pp. 56. 1859. (From the author.)

The Present State of Microscopical Science, Medically Considered. By John H. Packard, M.D. Reprint. 8vo., pp. 43. Philadelphia: J. B. Lippincott & Co., 1859. (From the author.)

On Recto-Vesical Lithotomy, with the Report of Cases in which this Method was successfully employed. By Louis Bauer, M.D., etc. Reprint, pp. 14. New York, 1859. (From the author.)

Description of a New Hysterotome for the Cure of Dysmenorrhœa. By O. A. White, M.D. Reprint, pp. 7. Charleston, 1859. (From the author.)

Case of Hermaphrodism. By J. Mason Warren, M.D. Reprint, pp. 6. Philadelphia, 1859. (From the author.)

The Brooklyn City Hospital in 1858, and the Address. By T. C. Hutchinson, M.D., etc. Pamphlet, 8vo., pp. 38. Brooklyn, 1859. (From the author.)

Hygiene. Being the Substance of the "Charge" given February, 1859, to the Graduating Class in the Medical Department of the University of Buffalo. By Frank Hastings Hamilton, M.D., etc. 8vo., pamphlet, pp. 15. New York, 1859. (From the author.)

### FOREIGN.

The Action of Medicines in the System. By Frederick William Headland, M.D., etc. 3d edition. 8vo., pp. 463. Philadelphia: Lindsay & Blakiston, 1859. (From the publishers.)

Urinary Deposits; their Diagnosis, Pathology, and Therapeutical Indications. By Golding Bird, M.D., etc. Edited by Edmund Birkett, M.D., etc. A New American, from the Fifth London Edition. 8vo. pp. 382. Philadelphia: Blanchard & Lea, 1859. (From the publishers.)

A History of the Discovery of the Circulation of the Blood. By P. Flourens. Translated from the French by J. C. Reeve, M.D., etc. pp. 175. Cincinnati: Rickey, Mallory & Co., 1859.

The Treatment of Ulcers and Cutaneous Eruptions on the Leg, without Confinement. By Henry T. Chapman, F.R.S., etc. 3d edition. Post 8vo., pp. 161. London: Churchill, 1859.

The Pathology of Tuberculous Bone. By C. Black, M.D., etc. pp. 40. Edinburgh: Sutherland & Knox, 1859.

A Treatise on Vital Causes. By James Newton Heale, M.D., etc. 8vo. London: Churchill, 1859.

Lectures on Pathological Anatomy, etc. By Samuel Wilks, M.D., etc. 8vo., pp. 480. London: Longman, Brown & Co., 1859.

Epiphora, or Watery Eye; its Successful Treatment by the New Method of Dilatation. By James Vose Solomon, F.R.C.S., etc. 8vo. London: Churchill, 1859.

On the Treatment of the Epidemic Cholera. By Joseph Ayre, M.D. 8vo. London: Churchill, 1859.

Notes on Hospitals. By Florence Nightingale. 8vo., pp. 108. John W. Parker & Son: London, 1859.

Medical Sketches in Austria, Prussia, and Italy. By James G. Hildige, F.R.C.S.I., etc. pp. 86. Dublin: Fannin & Co., 1859.

